# Changes in the Left Ventricular Eicosanoid Profile in Human Dilated Cardiomyopathy

**DOI:** 10.3389/fcvm.2022.879209

**Published:** 2022-05-19

**Authors:** Deanna K. Sosnowski, K. Lockhart Jamieson, Ahmed M. Darwesh, Hao Zhang, Hedieh Keshavarz-Bahaghighat, Robert Valencia, Anissa Viveiros, Matthew L. Edin, Darryl C. Zeldin, Gavin Y. Oudit, John M. Seubert

**Affiliations:** ^1^Faculty of Pharmacy and Pharmaceutical Sciences, University of Alberta, Edmonton, AB, Canada; ^2^Department of Medicine, Mazankowski Alberta Heart Institute, Edmonton, AB, Canada; ^3^Mazankowski Alberta Heart Institute, Edmonton, AB, Canada; ^4^Department of Pharmacology, Faculty of Medicine and Dentistry, University of Alberta, Edmonton, AB, Canada; ^5^Division of Intramural Research, National Institute of Environmental Health Sciences, National Institutes of Health (NIH), Durham, NC, United States

**Keywords:** dilated cardiomyopathy (DCM), eicosanoids, mitochondria, soluble epoxide hydrolase (sEH), CYP450

## Abstract

**Objective:**

Metabolites derived from *N*−3 and *N*−6 polyunsaturated fatty acids (PUFAs) have both beneficial and detrimental effects on the heart. However, contribution of these lipid mediators to dilated cardiomyopathy (DCM)-associated mitochondrial dysfunction remains unknown. This study aimed to characterize DCM-specific alterations in the PUFA metabolome in conjunction with cardiac mitochondrial quality in human explanted heart tissues.

**Methods:**

Left ventricular tissues obtained from non-failing control (NFC) or DCM explanted hearts, were assessed for *N*−3 and *N*−6 PUFA metabolite levels using LC-MS/MS. mRNA and protein expression of CYP2J2, CYP2C8 and epoxide hydrolase enzymes involved in *N*−3 and *N*−6 PUFA metabolism were quantified. Cardiac mitochondrial quality was assessed by transmission electron microscopy, measurement of respiratory chain complex activities and oxygen consumption (respiratory control ratio, RCR) during ADP-stimulated ATP production.

**Results:**

Formation of cardioprotective CYP-derived lipid mediators, epoxy fatty acids (EpFAs), and their corresponding diols were enhanced in DCM hearts. These findings were corroborated by increased expression of CYP2J2 and CYP2C8 enzymes, as well as microsomal and soluble epoxide hydrolase enzymes, suggesting enhanced metabolic flux and EpFA substrate turnover. DCM hearts demonstrated marked damage to mitochondrial ultrastructure and attenuated mitochondrial function. Incubation of fresh DCM cardiac fibers with the protective EpFA, 19,20-EDP, significantly improved mitochondrial function.

**Conclusions:**

The current study demonstrates that increased expressions of CYP-epoxygenase enzymes and epoxide hydrolases in the DCM heart correspond with enhanced PUFA-derived EpFA turnover. This is accompanied by severe mitochondrial functional impairment which can be rescued by the administration of exogenous EpFAs.

## Introduction

Dietary lipids play a critical role in the maintenance of cardiac function and homeostasis (1). Humans obtain their majority of N-3 and N-6 polyunsaturated fatty acids (PUFAs) from dietary sources such as vegetable oils, sunflower and flaxseed oil, and fish (1). Once consumed, PUFAs are esterified into cell membranes and free fatty acids can be metabolized by cyclooxygenase (COX), lipoxygenase (LOX), and cytochrome P450 (CYP)-epoxygenase enzyme systems into a myriad of biologically active lipid mediators called eicosanoids (1). Eicosanoids function on the cellular and molecular level as signaling molecules and modifiers of biological processes such as inflammation (1, 2). Perturbations in the lipid mediator metabolite profile underly numerous cardiovascular diseases (CVD), highlighting its important role in CVD development and progression (3, 4). Changes in the PUFA metabolite composition and enzyme systems have been characterized in hearts from patients with ischemic cardiomyopathy and unspecified non-ischemic heart failure (3, 5, 6). However, the *N*−3 and *N*−6 PUFA metabolome has not been specifically reported in hearts from male and female dilated cardiomyopathy (DCM) patients.

DCM is a form of non-ischemic heart failure characterized by dilation of the ventricles accompanied by systolic dysfunction and reduced cardiac output (7). In addition to ventricular remodeling and fibrosis, impairment in cardiac mitochondrial function and bioenergetics are hallmarks of this disease. However, the underlying pathophysiological mechanisms of DCM development and progression remain poorly understood (7, 8). To date, only a limited number of studies have examined eicosanoid profiles and the expression of key enzyme systems in cardiac tissues from human DCM patients (9, 10).

In this brief report, we characterize alterations in the *N*−3 and *N*−6 PUFA-derived eicosanoid metabolome in left ventricular tissue obtained from human explanted DCM and non-failing control (NFC) hearts. We correlate eicosanoid levels with CYP-epoxygenase and epoxide hydrolase enzyme expression along with biomarkers of cardiac mitochondrial quality. We provide proof-of-concept evidence demonstrating direct application of exogenous eicosanoids from CYP450-dependent metabolism improve DCM cardiac mitochondrial function. Overall, these identified alterations in the eicosanoid metabolome open the door for further investigation of lipid mediators as pharmacological targets for the treatment of DCM.

## Materials and Methods

### Patient Clinical Data and Donor Heart Tissue Procurement

Left ventricular (LV) biopsies from male and female NFC donors with no history of CVD were obtained from the Human Organ Procurement and Exchange (HOPE) at the University of Alberta. Additionally, biopsies from DCM patients with end-stage heart failure were procured as part of the Human Explanted Heart Program (HELP) during transplant procedures at the Mazankowski Alberta Heart Institute (11). Tissue collection followed protocols approved by the Health Research and Ethics Board of the University of Alberta. Both the demographic and clinical data from NFC and DCM patients is summarized in Table 1. Data from DCM patients corresponds to clinical assessment prior to when heart transplant was performed.

**Table 1:** Demographic and clinical parameters of patients acquired from electronic medical records.

**NFC**			
	**Male** ***n*** **=** **5**	**Female** ***n*** **=** **6**	***P*** **-Value**				
**Demographics**							
Age, yrs	38 (27–38)	51 (44–56)	0.11
BMI, kg/m^2^	23.1 (21.9–24.4)	25.5 (24.2–27.6)	0.20
**Echocardiography**
LVEF (%)	45 (40–50)	55 (43–60)	0.35
**DCM**							
	**Male** ***n*** **=** **12**	**Female** ***n*** **=** **6**	***P*** **-Value**		**Male** ***n*** **=** **12**	**Female** ***n*** **=** **6**	***P*** **-Value**
**Demographics**				**Functional class**			
Age, yrs	52 (47–56)^#^	55 (44–55)	0.91	NYHA II	1 (8.3)	1 (16.7)	>0.99
BMI, kg/m^2^	24.7 (22.6–25.2)	25.8 (22.1–32.6)	0.55	NYHA III	7 (58.3)	2 (33.3)	0.62
**Laboratory tests**				NYHA IV	4 (33.3)	3 (50.0)	0.63
Hemoglobin, g/L	119 (113–134)	108 (101–113)	0.12	**Comorbidities**			
eGFR, mL/min/m^2^	57 (51–62)	56 (44–61)	0.77	Atrial fibrillation	2 (16.7)	0	0.53
**Medications**				Kidney disease	9 (75.0)	4 (66.7)	>0.99
ACE inhibitor	7 (58.3)	6 (100)	0.11	Diabetes mellitus	2 (16.7)	1 (16.7)	>0.99
ARB	2 (16.7)	2 (33.3)	0.57	Hypertension	1 (8.3)	1 (16.7)	>0.99
β-blocker	9 (75.0)	6 (100)	0.51	COPD/Asthma	2 (16.7)	4 (66.7)	0.11
Loop diuretics	11 (91.7)	4 (66.7)	0.25	Dyslipidemia	1 (8.3)	0	>0.99
MRA	8 (66.7)	4 (66.7)	>0.99	Liver disease	3 (25.0)	2 (33.3)	>0.99
Antiplatelet	7 (58.3)	4 (66.7)	>0.99	**Devices**			
Anticoagulant	8 (66.7)	6 (100)	0.25	AICD/ICD	5 (41.7)	3 (50.0)	>0.99
Statin	3 (25)	2 (33.3)	>0.99	BiV-ICD	2 (16.7)	1 (16.7)	>0.99
Antiarrhythmics				CRT	2 (16.7)	3 (50.0)	0.27
Class I	1 (8.3)	2 (33.3)	0.25	**Vitals**			
Class III	3 (25.0)	5 (83.3)	0.043^*^	Heart rate, bpm	96 (74–103)	95 (81–99)	0.86
**Echocardiography**				Systolic BP, mmHg	101 (91–116)	106 (93–120)	0.75
LVEF (%)	18 (13–20)	20 (14–29)	0.68	Diastolic BP, mmHg	64 (56–73)	67 (58–67)	>0.99

Descriptive data is expressed as median (interquartile range, IQR) and analyzed using non-parametric Mann Whitney U-test. Categorical data is expressed as absolute number (%) and analyzed using standard chi square with Fisher's exact test (each individual patient = n, male NFC n = 5, male DMC n = 12, female NFC n = 6, female DCM n = 6, ^*^p < 0.05 DCM male vs. DCM female, ^#^p < 0.05 vs. NFC male).

### Eicosanoid Metabolome Profile

Levels of eicosanoids and their metabolites were quantified by LC-MS/MS similar to previous descriptions (12). Briefly, LV tissue was homogenized in Hank's Balanced Salt Solution containing 1 μM trans-4-[4-(3-Adamantan-1-yl-ureido)-cyclohexyloxy]-benzoic acid to a concentration of 100 mg/ml. 3 ng of internal standards (11,12-EET-d11, 11,12-DHET d11, 15-HETE-d8, and PGE_2_-d9; Cayman Chemical, Ann Arbor, MI) were added to 10 mg heart tissue (100 μl of homogenate) which was extracted with 600 μl ethyl acetate. After mixing and centrifugation, the organic phase was transferred to glass tubes containing 6 μl of 30% glycerol in methanol (Sigma) and dried under vacuum centrifugation. Samples were reconstituted in 50 μl of 30% ethanol and 10 μls was injected into the Agilent 1200 Series capillary HPLC (Agilent Technologies, Santa Clara, CA, USA) system and separated using the Phenomenex Luna C18(2) column (5 μm, 150 × 1 mm; Phenomenex, Torrance, CA, USA). Two mobile phases were used: A; 85:15:0.1 water:acetonitrile:acetic acid and B; 70:30:0.1 acetonitrile:methanol:acetic acid. All samples were run in triplicate. Detection of components in the eluted samples were done by negative ion electrospray ionization tandem mass spectrometry using multiple reaction monitoring by an MDS Sciex API 3000 equipped with a TurboIonSpray source (Applied Biosystems). Analytes were quantified using Analyst 1.5.1 software (Sciex) calibrated to standard curves derived from injections of commercial standards for each oxylipin (Cayman) (12).

### Protein Expression and Immunoblot Analysis

Heart tissue was homogenized in buffer containing Tris-HCl 10 mM, EDTA 1 mM, sodium orthovanadate 1 mM, sodium fluoride 1 mM, aprotinin 10 μg/L, leupeptin 2 μg/L, pepstatin 100 μg/L and sucrose 250 mM. Whole tissue homogenate was centrifuged at 700 × g for 10 min. The supernatant was collected and spun at 10,000 × g for 20 min to isolate the crude mitochondrial pellet and the supernatant was centrifuged at 100,000 × g for 1 h to pellet the microsomal fraction isolate the cytosol (3). Briefly, 30 ug of protein from each sample was resolved onto gradient gels (4–15%) and transferred onto a 0.2 μm PVDF membrane. Primary antibodies were used at a concentration of 1:1,000 (sEH, sc25797; GAPDH, cs5174; mEH, sc135984; CYP2J2, ABS1605; CYP2C8, ab88904; β-Actin, sc47778; VDAC, ab14734). Densitometry was performed with ImageJ software [National Institutes of Health (NIH), USA] and protein expressions were normalized to their respective loading controls.

### RNA Extraction and Microarray Analysis

Tissue RNA was extracted for analysis using the RNeasy Mini Kit (Qiagen Cat74104) (13, 14). Purity of RNA was confirmed by measuring sample absorbance at 260 and 280 nm wavelength. Microarray was performed using PrimeView Human Gene Expression Array (ThermoFisher Cat902487) according to the manufacturer's protocol. Changes in mRNA transcripts were assessed by TaqMan reverse PCR using fluorescent probes (14, 15). Expression of transcripts of interest were normalized to 18S mRNA levels in the sample.

### Assessment of Cardiac Mitochondrial Quality

Transmission electron microscopy (TEM) was used to assess mitochondrial morphology and ultrastructure in myocardial tissue (16). Slices were imaged within 1 week of staining at 60 kV using a transmission electron microscope (Hitachi H-7650 TEM, Hitachi High-Technologies Canada, Inc) with a 16-megapixel EMCCD camera (XR111, Advanced Microscopy Technique, MA, USA) at the University of Alberta, Faculty of Medicine and Dentistry, EM Core Facility. ImageJ was used to assess mitochondrial circularity index and the presence and severity of inclusion bodies and cristae morphology through a semi-quantitative scoring method (17). Images were randomly scored in triplicate by two blinded, independent investigators.

Complex I (NADH:ubiquinone oxidoreductase), complex II (succinate dehydrogenase) and citrate synthase activities were determined in mitochondrial tissue fractions as previously described (3, 18). Briefly, individual cuvettes containing sample and substrates for complex I (NADH 100 μM, ubiquinone 60 μM), complex II (succinate 20 mM, DCPIP 80 μM, decylubiquinone 50 μM) or citrate synthase (DTNB 100 μM, acetyl coenzyme A 300 μM) were prepared. Specific inhibitors for complex I (rotenone 10 μM) and complex II (malonate 10 mM) were added in a parallel assay to account for non-specific complex activity. Absorbance (complex I, 340 nm; complex II, 600 nm; citrate synthase, 412 nm) was monitored spectrophotometrically over 2–3 min. Complex activity was calculated using linear absorbance, substrate extinction coefficient, sample volume, and protein concentration.

A bioluminescent assay kit (Abcam, ab65313) was used to quantify ATP and ADP levels in crude mitochondrial fractions from LV tissues. Samples were combined with reaction buffer containing ATP-monitoring enzyme and nucleotide releasing buffer. Luminescence was measured to determine levels of tissue ATP. Then, ADP-converting enzyme was added to each sample to determine ADP levels. Finally, the ratios of ATP to ADP levels were calculated for NFC and DCM male and female hearts.

Determination of mitochondrial oxygen consumption was performed using a Clark electrode connected to an Oxygraph Plus recorder (Hansatech Instruments Ltd., Norfolk, UK). Non-frozen, fresh cardiac fibers were isolated from hearts within 30 min post-transplant. Fibers were permeabilized with isolation buffer containing saponin (100 μg/mL), and added to the 30°C respiration chamber containing 2 mL of respiration buffer as previously described (19, 20). Basal and ADP-stimulated (0.5 mmol/L) respiratory rates using malate (5 mmol/L) and glutamate (10 mmol/L) as substrates were recorded followed by the addition of vehicle or 19,20-EDP (1 μM) treatment. Respiratory control ratio (RCR) was calculated as the ratio between basal and ADP-stimulated respiration rates to indicate efficiency of ATP production.

### Statistics

Descriptive clinical data were expressed as the median (interquartile range, IQR) and analyzed using the non-parametric Mann Whitney u-test. Categorical clinical data were expressed as absolute number (%) and analyzed using standard chi square with Fisher's exact test. Molecular and biochemical data were analyzed using one-way ANOVA with Tukey's multiple comparisons test. A *p* < 0.05 was considered to be significant.

## Results

### Patient Characteristics and Clinical Data

Left ventricular biopsies were obtained from male and female DCM patients with a median age of 52 and 55 years old, respectively (Table 1). Median left ventricular ejection fraction was 18–20%, indicating severe systolic dysfunction and presence of heart failure. Degree of heart failure ranged from NYHA functional class II–IV. More than 80% of male and female patients comprised NYHA functional class III and IV, indicating that the majority of DCM patients were severely symptomatic and markedly impaired due to heart failure. Nearly half of male and female patients had implanted cardiac rhythm devices including cardioverter-defibrillators (ICD) and cardiac resynchronization therapy (CRT) pacemakers. All female DCM patients were taking angiotensin converting enzyme (ACE) inhibitors, beta blockers, and anticoagulants. The majority of male and female patients were also prescribed loop diuretics, mineralocorticoid receptor antagonists (MRA), and antiplatelets such as acetylsalicylic acid (ASA). A significantly larger proportion of female patients were taking class III antiarrhythmic medications compared to their male counterparts. The majority of DCM patients also suffered from existing comorbidities including COPD, asthma, liver disease, and hypertension. Kidney disease and renal dysfunction were the most prevalent comorbid conditions in both male and female patients. Lastly, both male and female DCM patients had similar laboratory and vital parameters.

NFC LV tissue was obtained from donor hearts deemed not suitable for transplant. Available clinical data was limited for NFC patients. Patients had no known history of CVD. The median age was 38 years for males and 51 years for females, but this did not constitute a statistically significant difference. Median male and female left ventricular ejection fractions were 45% and 55%, respectively. There were no other significant differences between parameters from male and female NFC patients.

### DCM Hearts Have Perturbations in the *N*−3 and *N*−6 PUFA Eicosanoid Metabolome

Lipids are critical components of cellular structure and signaling and are required to maintain homeostasis and proper function of the heart (1, 9). Alterations in the composition of metabolites derived from *N*−3 and *N*−6 PUFAs can impact CVD pathophysiology. Using LC-MS/MS, we profiled changes in COX, LOX, and CYP450-epoxygenase metabolic products derived from *N*−3 and *N*−6 PUFAs in DCM human heart tissue compared to NFC (Table 2). COX-dependent metabolites of arachidonic acid (AA) with pro-inflammatory properties such as prostaglandins (PG), PGD_2_, and PGE_2_, were significantly increased in DCM hearts compared to NFC tissues. While the prothrombotic and vasoconstrictive COX-derived metabolite, thromboxane B_2_ (TXB_2_) was lower in the hearts of DCM patients. Oxidative metabolism of the *N*−6 PUFA, linoleic acid (LA) produces hydroxyoctadecadienoic acids (HODEs). Known biological effects of HODEs have been associated with inflammatory-driven atherosclerotic plaque progression, but have not been evaluated in the context of DCM (21). Levels of 9-HODE and 13-HODE were strikingly elevated in DCM hearts highlighting necessary future investigation into the implication of HODEs in DCM. Epoxygenation of *N*−3 and *N*−6 PUFAs by CYP450 isoenzymes yield a class of lipid mediators broadly referred to as epoxy fatty acids (EpFAs) (1). EpFAs have established cardioprotective, anti-inflammatory, and mitochondrial protective functions (1). Regioisomers, 9,10-, and 12,13- epoxyoctadecenoic acids (EpOMEs), and 8,9-, 11,12-, and 14,15-epoxyeicosatrienoic acids (EETs) derived from N-6 PUFAs such as LA and AA, were elevated in DCM hearts. Whereas, *N*−3 PUFA epoxygenation products including 19,20-epoxydocosapentaenoic acid (EDP) remained unaltered, suggesting preferential epoxidation of lipids containing their double bond at the omega-6 position.

**Table 2:** *N*−3 and *N*−6 oxylipin profile (ng/g tissue) from LV heart biopsies measured by LC-MS/MS.

	**Male**		**Female**	
	**NFC**	**DCM**	**NFC**	**DCM**
**COX-dependent**
6-keto PGF_1α_	5.4 ± 2.1	2.4 ± 0.9	5.7 ± 1.1	2.7 ± 2.3
TXB_2_	0.2 ± 0.06	0.08 ± 0.02^*^	0.2 ± 0.06	0.05 ± 0.04
PGF_2α_	5.1 ± 3.1	7.7 ± 4.4	1.1 ± 0.5	2.6 ± 0.4
PGE_2_	6.0 ± 1.5	27.5 ± 4.3^*^	9.0 ± 2.7	13.8 ± 4.8
PGD_2_	10.6 ± 2.9	43.1 ± 4.6^*^	16.5 ± 4.4	23.9 ± 8.3
8isoPGF_2α_	1.0 ± 0.09	1.8 ± 0.3	1.1 ± 0.09	1.1 ± 0.2
**LOX-dependent**
9-HODE	159.5 ± 25.0	1,074.4 ± 207.3^*^	359.4 ± 154.5	675.7 ± 417.9
13-HODE	282.5 ± 37.6	1,488.7 ± 278.6^*^	542.2 ± 204.6	970.3 ± 527.1
5-HETE	26.8 ± 6.7	35.5 ± 8.0	36.0 ± 5.8	17.6 ± 5.1
11-HETE	29.6 ± 7.6	33.2 ± 6.4	33.8 ± 5.8	18.2 ± 8.2
12-HETE	40.9 ± 12.4	34.0 ± 7.2	45.6 ± 7.9	18.5 ± 8.1
15-HETE	39.9 ± 9.9	53.1 ± 13.2	48.8 ± 8.0	23.7 ± 9.7
**CYP-dependent**
9,10-EpOME	63.7 ± 22.1	411.6 ± 71.6^*^	92.4 ± 36.0	245.8 ± 87.4
12,13-EpOME	17.5 ± 3.9	106.2 ± 20.7^*^	28.7 ± 9.9	61.1 ± 24.0
8,9-EET	11.6 ± 1.4	20.9 ± 2.5^*^	12.6 ± 1.9	12.8 ± 3.3
11,12-EET	5.6 ± 0.9	14.0 ± 2.7^*^	6.7 ± 0.9	5.0 ± 2.0^#^
14,15-EET	8.8 ± 1.0	13.9 ± 1.3^*^	10.9 ± 1.2	8.5 ± 2.23
19,20-EDP	47.9 ± 14.3	47.8 ± 11.8	35.6 ± 2.3	35.94± 9.6
**sEH/mEH-dependent**
9,10-DiHOME	8.7 ± 2.3	74.0 ± 10.8^*^	12.0 ± 3.8	37.9 ± 10.9^*^
12,13-DiHOME	4.9 ± 0.7	17.8 ± 3.5^*^	6.1 ± 1.6	16.3 ± 4.7^*^
8,9-DHET	0.8 ± 0.1	1.6 ± 0.2^*^	0.7 ± 0.1	1.2 ± 0.4
11,12-DHET	0.8 ± 0.08	0.9 ± 0.1	0.9 ± 0.1	1.0 ± 0.4
14,15-DHET	0.6 ± 0.08	0.8 ± 0.1	0.5 ± 0.07	0.5 ± 0.04
**EpFA:diol ratios**
9,10-EpOME:DiHOME	7.0 ± 0.8	5.9 ± 0.8	7.4 ± 0.7	6.5 ± 1.1
12,13-EpOME:DiHOME	3.5 ± 0.5	7.2 ± 1.3	5.1 ± 1.4	3.7 ± 0.7
8,9-EET:DHET	17.4 ± 2.8	15.1 ± 2.5	20.4 ± 3.6	12.5 ± 5.1
11,12-EET:DHET	7.9 ± 1.7	17.8 ± 2.9^*^	8.3 ± 1.0	5.5 ± 2.1^#^
14,15-EET:DHET	15.2 ± 3.4	21.1 ± 2.9	23.5 ± 3.1	15.6 ± 3.7

Data were analyzed using ordinary one-way ANOVA with Tukey's multiple comparisons test and are expressed as mean ± SEM (male NFC n = 5, male DCM n = 10, female NFC n = 4, female DCM n = 3, ^*^ p < 0.05 vs. NFC, ^#^p < 0.05 vs. male counterpart).

Bioactive EpFAs can undergo hydrolysis by endogenous epoxide hydrolase enzymes to their corresponding vicinal diol products (1). EpOME-derived diols, 9,10- and 12,13-dihydroxyoctadecanoic acids (DiHOMEs) and 8,9-dihydroxyeicosatrienoic acid (DHET) derived from 8,9-EET were increased in DCM tissue. With respect to the heart, current evidence suggests EpFA-derived diol metabolites possess weaker biological activity compared to their epoxygenated precursors (1). Hence, the enhanced formation of N-6 PUFA-derived EpFAs are likely followed by their subsequent hydrolysis in the hearts of DCM patients.

Tissues from male and female DCM hearts exhibited similar trends in alterations to their eicosanoid metabolome compared to NFC. Interestingly, these changes were more prominent in male DCM hearts compared to female counterparts. Furthermore, EpFA:diol ratios differed between male and female DMC patients. The majority of ratios were increased in males and decreased in females, with significant differences between 11,12-EET:DHET. Hence, there is the potential impact of sex-dependent mechanisms in the interpretation of these findings.

### Expression of Key Enzymes Corroborate Alterations in the DCM Eicosanoid Metabolome

**Figure 1:**
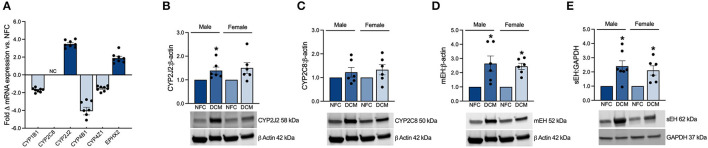
**(A)** Fold change in mRNA expression of key enzymes in DCM LV tissue vs. NFC based on microarray analysis (NFC *n* = 8 and DCM *n* = 8; NC = no change). Western immunoblot quantification and representative blots showing protein expression of **(B)**, CYP2J2; **(C)**, CYP2C8; **(D)**, mEH; **(E)**, sEH enzymes in microsomal and cytosolic heart tissue fractions. Data are expressed as mean ± SEM and analyzed using one-way ANOVA with Tukey's multiple comparisons test (each individual patient heart = *n*, male NFC *n* = 6, female NFC *n* = 6, male DCM *n* = 6, female DCM *n* = 6, *p < 0.05 vs. NFC). The same loading control image is used for illustrative purposes in **(B–D)**.

CYP2J2 is abundantly expressed in the cells of the heart and its vasculature (5, 22, 23). CYP2J2 plays a predominant role in the metabolism of endogenous *N*-3 and *N*-6 PUFAs, generating numerous EpFAs. Currently, its role in CVD and cardiac drug metabolism is rapidly advancing (5, 22, 24). CYP2C8 is another isoform also involved in cardiac lipid metabolism, but to a lesser extent than CYP2J2 (22). Soluble epoxide hydrolase (sEH) and microsomal epoxide hydrolase (mEH) are responsible for the hydrolysis of EpFAs to their less-active diol metabolite products. mRNA expression of *CYP2J2* and *EPHX2* (sEH) were upregulated by 3.5 and 1.9-fold, respectively, while *CYP2C8* remained unchanged in DCM hearts (Figure 1A). Protein expressions of CYP2J2 and CYP2C8 were drastically increased in male and female DCM hearts, consistent with the enhanced production of EpFA metabolite products (Figures 1B,C). Expression of sEH and mEH were also significantly increased in male and female DCM tissues, corresponding to the enhanced formation of diols in the DCM heart (Figures 1D,E). Together, these data suggest enhanced enzymatic formation of EpFAs and subsequent turnover to diol metabolites, potentially impacting their availability to exert their bioactive effects.

### Metabolome Alterations Correspond With Mitochondrial Dysfunction

**Figure 2:**
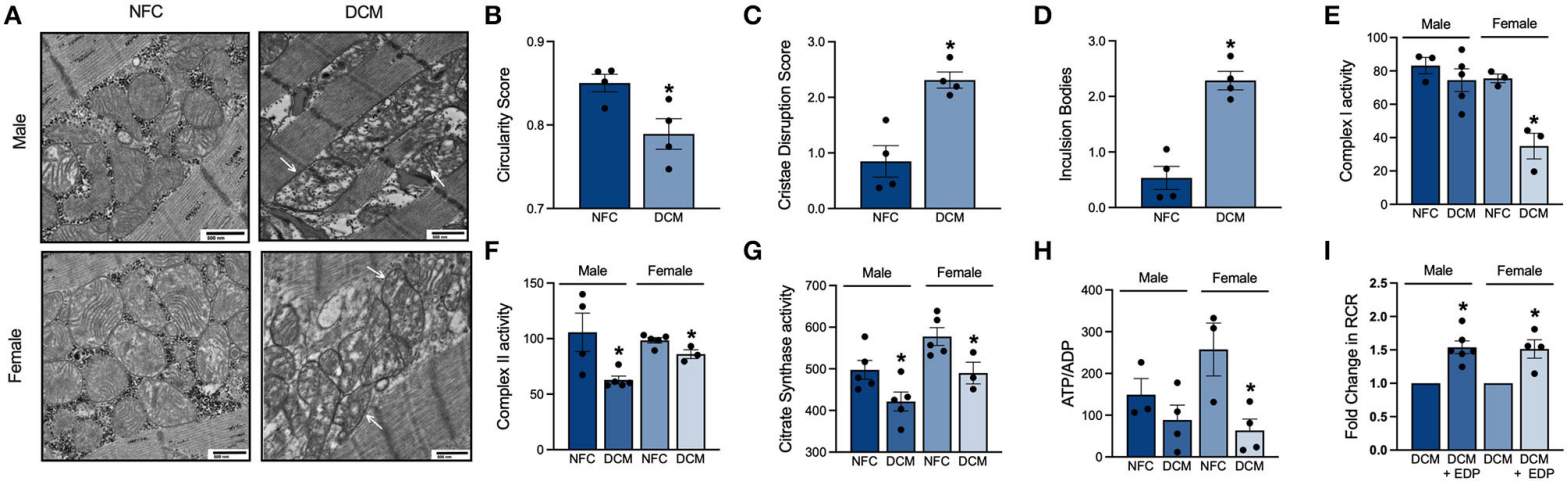
**(A)** Representative images of mitochondrial morphology and distribution in heart slices by transmission electron microscopy. White arrows indicate damaged mitochondria in DCM heart slices (10,000× magnification, scale bar = 500 nm). **(B)** Mitochondrial circularity score. **(C)** Mitochondrial cristae disruption score. **(D)** Average number of mitochondrial inclusion bodies. Specific enzymatic activities of mitochondrial electron transport chain **(E)** complex I; **(F)** complex II; and **(G)** citrate synthase in nmol/(min*mg) protein. **(H)** Relative ATP to ADP ratio in LV tissue. **(I)** Mitochondrial oxygen consumption represented by the respiratory control ratio (RCR) in basal and ADP-stimulated cardiac fibers. Data are expressed as mean ± SEM and analyzed using one-way ANOVA with Tukey's multiple comparisons test (each individual patient heart = *n*, male NFC *n* = 6, female NFC *n* = 6, male DCM *n* = 6, female DCM *n* = 6, *p < 0.05 vs. NFC).

Mitochondria are essential for energy production *via* fatty acid and glucose oxidation as well as calcium handling and cell signaling pathways within the cardiomyocyte (25, 26). Given the importance of mitochondria, we assessed their quality in NFC and DCM hearts. Using a 60 kV transmission electron microscope, distinct mitochondrial ultrastructural changes were visualized in the cardiomyocytes (Figure 2A). Mitochondria from DCM hearts exhibited a loss of circular morphology toward more elongated and irregular shapes (Figure 2B). Inner membrane cristae densities were reduced and disorganized compared to NFC (Figure 2C). As well, DCM mitochondria displayed enhanced presence of intraorganellar arrogates and deposition of inclusion bodies (Figure 2D), indicating impaired protein and macromolecular processing. The morphological changes were accompanied by significant decreases in activity of key electron transport chain enzymes, complex I (Figure 2E) and II (Figure 2F), as well as citrate synthase (Figure 2G). Furthermore, the ATP to ADP ratio in DCM hearts was significantly reduced (Figure 2H). Thus, mitochondrial function and energy production are compromised in DCM hearts.

Fresh, non-frozen, cardiac fibers from DCM, and NFC patients were isolated immediately following transplant to closely preserve the metabolic state and mitochondrial enzyme activity to *in vivo* levels. Permeabilized cardiac fibers from NFC hearts had an average respiratory control ratio (RCR) of 3.62. However, mitochondria from male and female DCM cardiac fibers had a diminished capacity for substrate utilization and ATP production, with RCRs of 1.51 and 2.30, respectively. Hence, there is a marked functional decline of cardiac mitochondria in DCM. Evidence demonstrates that the beneficial effects of EpFAs encompass their ability to limit mitochondrial dysfunction associated with CVD (27, 28). To investigate potential beneficial properties of EpFAs on mitochondrial function in DCM, exogenous 19,20-EDP was administered to permeabilized male and female DCM cardiac fibers. Importantly, administration of 19,20-EDP significantly improved mitochondrial RCR values compared to untreated DCM cardiac fibers (Figure 2I). These data demonstrate the protective role of exogenous EpFAs toward restoring mitochondrial function in DCM hearts.

## Discussion

PUFAs and their corresponding eicosanoid metabolite products impact the heart as well as the development and the progression of CVD (1, 29). In this study we demonstrated significant changes in the *N*−3 and *N*−6 PUFA-derived eicosanoid metabolome along with their associated metabolizing enzymes in human DCM and NFC left ventricular tissue. The eicosanoid metabolome alterations corresponded with severe mitochondrial dysfunction, which is a key underlying factor in DCM pathogenesis. Research pertaining to DCM poses a challenge to the scientific community due to its varying and often idiopathic etiologies (30). Therefore, defining molecular and cellular changes in human DCM hearts is critical toward understanding this disease.

*N*-3 and *N*-6 PUFAs such as eicosapentaenoic acid (EPA), docosahexaenoic acid (DHA), LA, and AA are largely obtained from the human diet (1). Endogenous COX, LOX, and CYP450 enzymes metabolize PUFA precursors to a plethora of bioactive lipid mediators, termed eicosanoids, which impact the cardiovascular system (1). Composition and levels of eicosanoids inside the body drastically affect metabolic profiles, phospholipid membrane composition, and cell signaling pathways both during homeostasis and during stressful biological conditions, such as disease (1, 31). DCM human heart tissue had enhanced production of pro-inflammatory eicosanoids, PGD_2_, PGE_2_, and 9- and 13-HODE, indicating a shift toward an enhanced inflammatory state in the DCM heart. Chronic inflammation underlies many forms of CVD (28). Administration of exogenous prostaglandins promotes a DCM-like phenotype of cardiomyocytes which further perpetuates prostaglandin secretion (32, 33). Thus, a self-sustaining pro-inflammatory environment exists within the human DCM heart.

The current study demonstrated alterations in CYP450 isoenzyme expression and EpFA formation in both male and female DCM hearts. Our observation showing increased CYP2J2 expression in DCM hearts contrasts with existing literature documenting CYP2J2 levels in CVD. Rather, the loss of CYP2J2 and CYP2C8 along with subsequent reduced EpFA formation has been associated with CVD. This association was first demonstrated in adults with coronary artery disease (34). Single nucleotide polymorphisms resulting in impaired transcription factor binding and thus the expression of the *CYP2J2* gene, are present at a higher frequency in individuals with angiographically documented coronary artery disease (34). In explanted left ventricular tissue from humans with ischemic heart disease, CYP2J2 and CYP2C8 levels were lower in the infarcted regions (3). This was accompanied by significantly reduced levels of the EpFA, 9,10-, and 12,13-EpOME, respectively (3). In a recent study assessing CYP2J2 expression in cardiac tissue from patients with unspecified non-ischemic cardiomyopathy there was a marked reduction in this enzyme (5). Furthermore, in a murine model of type 2 diabetes, CYP2J and CYP2C enzyme families were also downregulated in the heart which can disrupt the balance between cardioprotective EpFA and cardiotoxic metabolite counterparts (35). Hence, our findings are in contrast to the current literature investigating forms of CVD other than DCM.

Conversely, transgenic over-expression of human CYP2J2 in murine cardiomyocytes enhances EpFA production which preserves cardiac function in ischemia-reperfusion injury, diabetic cardiomyopathy, and attenuates arrythmia susceptibility in cardiac hypertrophy. Therefore, enhanced CYP2J2 expression confers a protective role in the diseased heart. Well-established evidence supports the cardioprotective, anti-inflammatory, and mitochondrial protective roles of EpFAs in CVD (36). Our current findings suggest increased CYP2J2 and EpFA levels potentially represent a compensatory endogenous mechanism in attempt to protect the failing DCM heart.

Upon formation from their *N*−3 and *N*−6 PUFA precursors, EpFAs are re-esterified into the plasma membrane of the cell (37). Cellular stress and injury can prompt the rapid release of membrane-bound EpFAs *via* phospholipase A_2_, making free EpFAs widely available for cellular use (37, 38). However, liberation also predisposes EpFAs to metabolism and inactivation by intracellular epoxide hydrolase enzymes. We showed that sEH and mEH enzyme levels were also increased in DCM hearts suggesting rapid-turnover of newly synthesized or liberated EpFAs. sEH is the primary epoxide hydrolase responsible for the metabolism of EpFAs throughout the body (39). mEH plays a less prominent role, but contributes to hydrolysis of residual EpFAs which escape sEH-dependent metabolism (39). Enhanced sEH expression and activity is associated with CVD, likely *via* the metabolism of cardioprotective and anti-inflammatory EpFAs in the heart (40). Furthermore, enhanced sEH activity due to *EPHX2* gene polymorphisms in humans was identified to be a potential risk factor for coronary heart disease (41). Inhibition of sEH activity *via* pharmacologic and genetic means has been employed as an experimental therapeutic strategy to preserve the pool of beneficial EpFAs in diseased and failing hearts (40). Therefore, enhanced CYP450-mediated formation of EpFAs may be insufficient to counterbalance the generation of pro-inflammatory COX and LOX eicosanoid metabolites in the DCM heart. EpFA protective effects are then further hindered by their accelerated removal *via* sEH and mEH-dependent hydrolysis to form diol products. Overall, this limits the length of their bioactive half-life and the extent to which they can protect the DCM heart. Therefore, both stimulating the release of membrane-bound EpFAs in conjunction with sEH enzymatic activity inhibition represents a viable approach to exploit the compensatory increase in CYP2J2 metabolic activity and delay the degradation of the protective EpFAs produced in the DCM heart.

Mitochondria make up more than 30% of the volume of the cardiomyocyte and supply more than 90% of ATP to the cell, highlighting the imperative role of these organelles in proper cardiac function (25). Impairment of mitochondrial quality and function, such as mitophagy, ATP production, and increased ROS generation are major contributors to the pathogenesis of DCM (8). Similarly, we demonstrated a dramatic decline to mitochondrial quality and function in human DCM hearts. CYP2J2 and CYP2C8-derived EpFAs, such as 14,15-EET and 19,20-EDP exert their cardioprotective effects, in part, by preserving cardiac mitochondrial integrity in CVD (27). We demonstrated that direct application of EpFAs to permeabilized DCM cardiac fibers can rescue mitochondrial function. Therefore, this supports the hypothesis that employing strategies to enhance endogenous EpFA effects could prove useful in DCM.

Some limitations to our study need to be considered with the data interpretation. Importantly, we were limited by the specific fresh cardiac tissue we received, as such, differences between the ages male of NFC and DCM hearts should be considered when assessing results. Hence, our observed differences in sEH-mediated metabolites in DCM male hearts, may in part, be influenced by aging. In aged male murine hearts, expression of sEH is elevated compared to young comparators (16). Other factors to consider include differences in patient medications. For example, use of aspirin as an antiplatelet therapy could contribute to the lower TXB_2_ levels in DCM hearts. Treatment with certain ACE inhibitors can prevent the breakdown of bradykinin and promote the formation of vasodilatory prostaglandins, which may explain higher levels of PGE_2_ in DCM hearts (42). Thus, highlighting the importance of understanding the effects of CVD medication toward the cardiac eicosanoid profile. In addition, we stratified the data by males and females, as sex is a critical and often under-emphasized determinant of CVD development, presentation and progression (43, 44). The impact of sexual dimorphism in our findings cannot be overlooked. DCM-associated increases in prostaglandins, HODEs, EpOMEs and EETs reached statistical significance in males but not in females. Underlying mechanisms of DCM pathogenesis such as apoptosis, fibrosis, and inflammatory cytokine production can be attributed to the male sex (45, 46). Some studies suggest differences in eicosanoid production and metabolism could explain sex-dependent differences in CVD risk and severity (47). Indeed, male DCM patients have been shown to have higher serum prostaglandin concentrations, which may suggest a greater involvement of pro-inflammatory eicosanoid signaling in male DCM patients (32). Apparent sex differences in EpFA:diol ratios may be attributed to higher expression of CYP2J2 in male DCM hearts, which did not reach significance in females. Whether this is a sex-dependent compensatory mechanism requires further investigation. Nonetheless, emphasis of these findings should be used as an opportunity to advocate for future research to understand sex-dependent mechanisms in DCM and eicosanoid biology.

In summary, these data demonstrate an altered PUFA eicosanoid metabolome, with a marked increase in the levels CYP450-epoxygenases and epoxide hydrolases in human DCM hearts. The presence of beneficial EpFAs was accompanied by the increased generation of their less potent diol metabolites. Thus, these findings suggest enhanced EpFA turnover, which may limit the extent of their cardioprotective actions. The inhibition of sEH activity in order to prolong the bioactive half-life of endogenously generated EpFAs along with the administration of exogenous EpFA compounds are potential therapeutic strategies to protect cardiac mitochondria in failing DCM hearts.

## Data Availability Statement

The original contributions presented in the study are included in the article/supplementary material, further inquiries can be directed to the corresponding author.

## Ethics Statement

The studies involving human participants were reviewed and approved by Health Research and Ethics Board of the University of Alberta. The patients/participants provided their written informed consent to participate in this study.

## Author Contributions

DS performed data acquisition, statistical analysis, and wrote the manuscript. KJ performed mitochondrial complex activity assays. AD performed mitochondrial respiration experiments. RV assisted with Western immunoblot experiments. HK-B assisted with data acquistion. HZ performed electron microscopy imaging and data acquisition, and patient clinical data collection. AV assisted with patient clinical data collection. ME, a member of DZ lab, performed LCMS/MS. GO is the director of the HELP and HOPE programs and was responsible for the procurement of LV tissue biopsies. JS is the primary investigator and was responsible for project design and manuscript preparation. All authors contributed to the article and approved the submitted version.

## Funding

This study was supported by a grant from the Heart and Stroke Foundation of Canada (HSFC GIA G-18-0021803 to JS). KJ was supported by a Graduate Studentship from Alberta Innovates-Health Solutions. AD was supported by an Alberta Innovates Graduate Studentship in Health Innovation, by the Izaak Walton Killam Memorial Scholarship, and by the BMO Financial Group Graduate Scholarship. HZ was supported by the China Scholarship Council (CSC) Award. This work was supported, in part, by CIHR project grant (PJT-156266 to GO) and the Intramural Research Program of the NIH, National Institute of Environmental Health Sciences (Z01 ES025034 to DZ).

## Conflict of Interest

The authors declare that the research was conducted in the absence of any commercial or financial relationships that could be construed as a potential conflict of interest.

## Publisher's Note

All claims expressed in this article are solely those of the authors and do not necessarily represent those of their affiliated organizations, or those of the publisher, the editors and the reviewers. Any product that may be evaluated in this article, or claim that may be made by its manufacturer, is not guaranteed or endorsed by the publisher.
